# Feeding with resistant maltodextrin suppresses excessive calorie intake in a high-fat diet, mediated by changes in mouse gut microbiota composition, appetite-related gut hormone secretion, and neuropeptide transcriptional levels

**DOI:** 10.3389/frmbi.2023.1149808

**Published:** 2023-04-21

**Authors:** Kaede Ito, Atsushi Haraguchi, Shuhei Sato, Masataka Sekiguchi, Hiroyuki Sasaki, Conn Ryan, Yijin Lyu, Shigenobu Shibata

**Affiliations:** Laboratory of Physiology and Pharmacology, School of Advanced Science and Engineering, Waseda University, Tokyo, Japan

**Keywords:** gut microbiota composition, appetite and food intake, appetite-related gut hormones, appetite-related neuropeptides, fecal microbiota transplantation (FMT), resistant maltodextrin

## Abstract

Consuming resistant maltodextrin (RMD) decreases food intake and increase appetite-related gut hormones, but the underlying mechanisms have remained unknown. Therefore, we aimed to elucidate the mechanisms underlying the effects of RMD feeding on food intake (appetite) using Institute of Cancer Research male mice fed with a high-fat diet (HFD-cellulose group) or HFD in which cellulose was replaced with RMD (HFD-RMD group). Feeding mice with an HFD-RMD for approximately 8 weeks inhibited excessive calorie intake and altered the gut microbiota composition. Excessive calorie intake was inhibited for several days in mice fed only with an HFD-cellulose and transplanted with fecal microbiota from the HFD-RMD group (FMT-HFD-RMD group). Moreover, in the HFD-RMD and FMT-HFD-RMD groups, serum active glucagon-like peptide (GLP)-1 and peptide tyrosine tyrosine (PYY) levels were significantly higher, and appetite-related neuropeptide gene transcription in the hypothalamus were significantly altered, compared with the HFD-cellulose and FMT-HFD-cellulose groups. These results suggested that the long-term RMD intake changed the gut microbiota composition, increased the GLP-1 and PYY secretion, and altered the appetite-related neuropeptide gene transcription in the hypothalamus, leading to suppressed excessive calorie intake in an HFD.

## 1 Introduction

The incidence of obesity is rapidly increasing worldwide and it is associated with myocardial infarction, diabetes, and non-alcoholic fatty liver, that are significant health problems (Huang, 2009). Causes of obesity include an imbalance between caloric intake and energy expenditure resulting from overeating and a lack of exercise (González-Muniesa et al., 2017). In addition, the gut microbiota has also been investigated as a cause of obesity. Indeed, a previous study in mice showed that transplantation of the gut microbiota of obese individuals made them obese (Turnbaugh et al., 2006). The prevalence of publications describing a relationship between the gut microbiota and metabolic diseases such as obesity, diabetes, and metabolic syndrome has recently increased (Amar et al., 2011; Ridaura et al., 2013; Suez et al., 2014; Cox et al., 2015). *Actinobacteria*, *Bacteroidetes*, *Firmicutes*, and *Proteobacteria* comprise ~ 90% of the gut microbiota and food, especially dietary fiber, easily influence the gut composition (Jandhyala et al., 2015). Studies of the relationship between appetite regulation and the gut microbiota composition (Han et al., 2021) have revealed that dietary habits influence the gut microbiota composition (Bäckhed et al., 2005; Cuevas-Sierra et al., 2019; Zhou et al., 2023). Therefore, we aimed to clarify whether dietary fiber could regulate food intake by altering the gut microbiota composition.

The brain-gut axis regulates appetite and eating behavior throughout an interdependent process mediated by the gut hormones signaling between the endocrine and nervous systems (Huda et al., 2006; Wren and Bloom, 2007; Steinert et al., 2017). Glucagon-like peptide-1 (GLP-1) and peptide tyrosine tyrosine (PYY) are gut hormones that stimulate satiety, decrease hunger, and promote meal cessation by signaling in the brain (Abou-Samra et al., 2022). Satiety-regulating gut hormones, such as active GLP-1 and PYY, positively correlate with activity in subcortical areas, such as the hypothalamus (Zanchi et al., 2017). For example, Active GLP-1 and PYY suppress appetite mediated by affecting appetite-related neuropeptides such as pro-opiomelanocortin (POMC; appetite suppression), cocaine- and amphetamine-regulated transcript (CART; appetite suppression), and neuropeptide Y (NPY; appetite stimulation) (Challis et al., 2003; Chen et al., 2007; Seo et al., 2008; Hill, 2010; Hamamah and Covasa, 2022). Intestinal enteroendocrine L-cells expressing G-protein coupled receptor (GPR) 41 or 43 bind butyrate and acetic acids, and secrete the active GLP-1 and PYY (Brown et al., 2003; Tazoe et al., 2009; Tolhurst et al., 2012; Billing et al., 2018; Hamamah and Covasa, 2022). Butyric and acetic acids are short-chain fatty acids (SCFAs) that are produced by the metabolic fermentation of water-soluble dietary fiber by the gut microbiota (Pluznick, 2016). Therefore, the present study focused on the gut microbiota of mice and water-soluble dietary fibers.

The mammalian gut microbiota comprises approximately 100 trillion gut bacteria. Various relationships between the gut microbiota and physical/pathological conditions have been uncovered using next-generation sequencing (Kostic et al., 2014; Merga et al., 2014; Marchesi et al., 2016). The relationship between appetite and SCFAs produced by the gut microbiota has been investigated. For example, dosing with intraperitoneally injected acetic acid or butyric acid given by intragastric gavage both suppressed the intake of a high fat diet (HFD) in mice after an overnight fast (Frost et al., 2014; Li et al., 2018).

Resistant maltodextrin (RMD) was the water-soluble dietary fiber in the present study. Dietary supplementation with RMD for one week reduces the calorie intake of an HFD to levels similar to those of a normal-fat diet (NFD) in rats and increases satiety in humans (Ye et al., 2015; Hira et al., 2018). In addition, these studies suggest that one week of RMD feeding increases the production levels of GLP-1 in the cecum of rats and plasma GLP-1 and PYY levels in humans (Ye et al., 2015; Hira et al., 2018). However, feeding rats with RMD for 7-8 weeks does not affect the intake of foods containing NFD and HFD (Hira et al., 2015; Hira et al., 2018). It is not known why the effects of RMD on food intake differ between short-term (one week) and long-term (7-8 weeks) RMD intake. Moreover, the mechanism by which RMD intake increases GLP-1 secretion and inhibits HFD-induced in calorie intake remains unknown.

We aimed to verify whether long-term RMD supplementation inhibits the increased calorie intake with HFD, changes the gut microbiota composition, and suppresses the increased calorie intake with HFD, and whether mice transplanted with the gut microbiota from mice supplemented with RMD show inhibited calorie intake increase with HFD. Moreover, to investigate the mechanism of the inhibitory effects on increased calorie intake due to HFD, we measured appetite-related gut hormones in the serum and neuropeptides in the hypothalamus.

## 2 Materials and methods

### 2.1 Animals

Seven-week-old Institute of Cancer Research (ICR) male mice (Tokyo Laboratory Animals Science Co. Ltd., Tokyo, Japan) were housed in an animal room at 22°C ± 2°C and 60% ± 5% humidity under a 12-h light/12-h dark cycle. Zeitgeber times (ZT) 0 (08:00) and 12 (20:00) were defined as when lights were turned on and off, respectively. The mice were habituated by feeding with an EF (EF; Oriental Yeast Co. Ltd., Tokyo, Japan) and tap water *ad libitum* for one week before starting experiments. In the present study, we used male ICR mice because some reports have suggested that ICR mice are a more appropriate model for metabolic research than C57BL/6J mice (Park et al., 2005; Park et al., 2006; Zhuhua et al., 2015; Narimatsu et al., 2022).

The experimental procedures complied with the Fundamental Guidelines for Proper Conduct of Animal Experiments and Related Activities in Academic Research Institutions (Ministry of Education, Culture, Sports, Science and Technology, Japan). The Committee for Animal Experimentation at Waseda University approved the study (2020-A039 and 2021-A072).

### 2.2 Diets

During experiments, mice fed with an NFD (MF; Oriental Yeast Co. Ltd.), HFD in which dietary fiber is only cellulose (water-insoluble dietary fiber), or HFD-RMD in which dietary fiber is RMD (water-soluble dietary fiber; Fibersol-2AG; Matsutani Chemical Industry Co., Hyogo, Japan) were given access to tap water *ad libitum*. The MF contained water-soluble and insoluble dietary fibers. The dietary fiber in AIN-93M (Oriental Yeast Co. Ltd.) is only cellulose. The HFD-cellulose comprised AIN-93M mixed with lard oil (Sigma-Aldrich Corp., St. Louis, MO, USA), and the HFD-RMD comprised AIN-93M in which cellulose was replaced with RMD and lard oil (**Table 1**). The reason why MF regarded as NFD was that AIN-93M contained only cellulose as dietary fiber and was considered inappropriate as a control food. The quantities of fiber, sugar, and moisture in the RMD were approximately 90%, 5%, and 5%, respectively. The average molecular weight of RMD is less than 2000 Da (Kishimoto et al., 2007).

**Table 1:** Ingredients of the diets [%].

	MF	HFD-cellulose	HFD-RMD
Casein	23.1^*^	11.2	11.2
L-cysteine		0.144	0.144
Corn starch	55.3^**^	37.25536	37.25536
Gelatinized corn starch		12.4	12.4
Sucrose		8	8
Soybean oil	5.1^***^	3.2	3.2
Lard oil		20	20
Tertiary butylhydroquinone		0.00064	0.00064
Cellulose powder	2.8^****^	4	
Resistant maltodextrin (RMD)			4
Mineral mix (AIN-93M-MX)	13.7^*****^	2.8	2.8
Vitamin mix (AIN-93VX)		0.8	0.8
Choline bitartrate		0.2	0.2
Sum	100	100	100

^*^crude protein, ^**^ crude carbohydrate, ^***^ crude fat, ^****^ crud dietary fiber, ^*****^ crud ash contents including moisture (7.9%).

### 2.3 Measurements of body weight and food intake

The mice were placed in individual cages in all experiments to monitor their food intake. The mice body weight was measured weekly. The food volume for each mouse was monitored weekly, calculated, and expressed as kcal/mouse/day. We measured food intake for one week before the fecal microbiota transplantation (FMT) in the FMT-donor mice, and for two days before, and daily at ZT10 after the FMT in the FMT-recipient mice.

### 2.4 Quantitation of SCFAs and lactic acid in cecal contents

Cecal content samples were collected under deep anesthesia. Subsequently, the cecal content samples were stored in sealable polypropylene microcentrifuge tubes at -80°C for subsequent analysis.

Cecal SCFAs and lactic acid might serve as markers of the status of the gut microbiota and environment. Lactic acid is the precursor of acetic and butyric acids. We therefore quantified SCFAs and lactic acid extracted from mouse cecal contents using gas chromatography and flame ionization detection (Shimadzu Co., Kyoto, Japan) as described (Huazano-García and López, 2013) with some modifications. Mouse cecal contents (approximately 50 mg) were suspended in 400 µL of diethyl ether (Fujifilm Wako Pure Chemicals, Osaka, Japan) containing 50 µL of sulfuric acid (Fujifilm Wako Pure Chemicals), and 200 µL of chloroform (Fujifilm Wako Pure Chemicals), then centrifuged at 13,000 × g for 30 s at room temperature. Thereafter, supernatants (1 µL) were injected into a 30 m × 0.25 mm InertCap Pure-WAX capillary column (df = 0.5 µm; GL Sciences, Tokyo, Japan), and the initial temperature was increased from 80°C to a final temperature of 200°C using helium as the carrier gas.

### 2.5 Fecal DNA extraction

Fecal samples were collected from the colon under deep anesthesia. Subsequently, the feces were stored in sealable polypropylene microcentrifuge tubes at -80°C for subsequent analysis.

We extracted gut microbiota DNA from mouse fecal samples as described (Nishijima et al., 2016). Feces (approximately 20 mg) in 20 mL of phosphate-buffered saline was filtered through a 100-µm nylon mesh (Corning Inc., Corning, NY, USA). The filtrate was centrifuged at 9,000 × g for 20 min at 4°C, then pellets were inverted with 800 µL TE10 buffer and 100 µL lysozyme (150 mg/mL; Sigma-Aldrich Corp.). Samples were incubated at 37°C for 1 h between each of these processes. Achromopeptidase (20 µL), proteinase K (50 µL) and 20% sodium dodecyl sulfate were sequentially added between incubations at 55°C. gut microbiota DNA was extracted using phenol: chloroform: isoamyl alcohol (25: 24: 1), 3 M sodium acetate, and isopropanol, and refined with 70% ethanol.

After fecal DNA extraction, DNA quantity was measured using the PicoGreen^®^ dsDNA Assay Kit (Invitrogen, USA) and diluted to 5 ng/µL with 10 mM Tris-HCl (pH 8.5).

### 2.6 Sequencing 16S rDNA

We analyzed 16S rDNA extracted from gut microbiota in fecal samples using an Illumina sequencing platform (Illumina MiSeq) according to the 16S Metagenomic Sequencing Library Preparation protocol (15044223 B).

The V3–V4 variable regions of the 16S rDNA gene were amplified using the polymerase chain reaction (PCR) with the respective forward and reverse (5′→ 3′) primers:

TCGTCGGCAGCGTCAGATGTGTATAAGAGACAGCCTACGGGNGGCCWGCAG

GTCTCGTGGGCTCGGAGATGTGTATAAGAGACAGGACTACHVGGGTATCTAATCC

Sequences of interest were amplified by PCR using 2.5 µL microbial DNA (5 ng/µL), 5 µL of each primer (1 µmol/L), and 12.5 µL 2 × KAPA HiFi HotStart Ready Mix (Kapa Biosystems Inc., Wilmington, MA, USA). The PCR conditions comprised 95°C for 3 min followed by 25 cycles of 95°C for 30 s, 55°C for 30 s, and 72°C for 30 s, followed by 72°C for 5 min and holding at 4°C. Amplicons were cleaned using AMPure XP beads (Beckman Coulter Inc., Brea, CA, USA) as described by the manufacturer. Purified DNA (5 µL) was amplified by index PCR using 5 µL of each Nextera XT Index Primer and sequenced using Nextera XT Index Kit v2 and an Illumina MiSeq (both from Illumina Inc.). Thereafter, index PCR products were amplified by PCR in 2 × KAPA HiFi HotStart Ready Mix (25 µL) and PCR-grade water (10 µL) under the following conditions: 95°C for 3 min followed by 8 cycles of 95°C for 30 s, 55°C for 30 s, and 72°C for 30 s, 72°C for 5 min, and hold at 4°C. The index PCR products were cleaned using AMPure XP beads (Beckman Coulter Inc.) as described by the manufacturer. Purity was verified using an Agilent 2100 Bioanalyzer with a DNA 1000 Kit (Agilent Technologies, Santa Clara, CA, USA). The concentration of the DNA library was adjusted to 4 nM, then the library sequenced using MiSeq Reagent Kit v3 (Illumina Inc.) and an Illumina MiSeq 2 × 300 bp platform as described by the manufacturer.

### 2.7 Analysis of 16S rDNA gene sequences

The 16S rDNA sequences were analyzed using the Quantitative Insights into Microbial Ecology (QIIME) pipeline version 1.9.1 (Caporaso et al., 2010). Filtered 16S rDNA sequences were assigned to operational taxonomic units based on having 97% similarity according to the UCLUST algorithm (Edgar, 2010), then compared with reference sequences in the Greengenes database (August 2013 version). We analyzed these sequences using QIIME to produce a taxonomy summary from the phylum to genus level, as well as alpha diversity (Simpson diversity index), and beta diversity. We also used weighted UniFrac distances for principal coordinate analysis.

### 2.8 Fecal microbiota transplantation

The fecal microbiota were transplanted as described (Wei et al., 2018). The FMT-recipient mice were fasted and housed in new individual cages for 1 h (ZT3−4). Polyethylene glycol (PEG) is an osmotic laxative that allows simple, rapid, and safe cleaning of the bowel (Mínguez et al., 2016). Bowel cleansing with PEG reduces ~ 90% of the total bacteria in the luminal and mucosal gut microbiota (Wrzosek et al., 2018). The intestinal tracts of FMT-recipient mice were washed using four oral administrations of PEG (200 µL, 425 g/L; Tokyo Chemical Industry Co., Ltd., Tokyo, Japan) at 20-min intervals 4 h before transplantation with gut microbiota from the FMT-donor mice. The FMT-donor mice were moved to new individual cages and fresh feces were collected. Fresh feces from each group were pooled in tubes. Subsequently, FMT-donor mice were returned to their individual home cages. The fresh feces (200 mg) from the FMT-donor mice were suspended in 5 mL PBS and finely ground using a spatula. The suspension was passed through a 100 µm nylon filter (Corning Inc.) and centrifuged at 5,500 × g for 5 min at 4°C. The supernatant was discarded, then the precipitate resuspended in PBS (200 µL; final concentration, 400 mg/mL) was orally administered to the FMT-recipient mice at ZT10. The FMT-recipient mice were then transferred to new cages and fed with HFD-cellulose.

### 2.9 Measurement of serum metabolic parameters

Terminal blood samples were collected from the retro-orbital sinus under deep anesthesia. Serum concentrations of active GLP-1 (Millipore Sigma, Burlington, MA, USA) and PYY (Fujifilm Wako Pure Chemicals) in mice were determined using ELISA kits as described by the manufacturer.

Active GLP-1 immediately undergoes limited degradation by dipeptidyl peptidase-IV (DPP-IV) in the blood and is transformed into inactive GLP-1. Therefore, to measured active GLP-1, DPP-IV inhibitor (final concentration, 50 µM) was placed in 0.6 mL tubes in advance. Blood samples were centrifuged at 600 × g for 10 min at 4°C within 10 min of collection. Each serum sample was divided into two tubes; tubes containing DPP-IV inhibitor for measurement of active GLP-1 and other tubes for measurement of PYY. All samples were stored in sealable polypropylene microcentrifuge tubes at -80°C.

### 2.10 Total RNA extraction and real-time reverse transcription PCR

Hypothalamus samples collected under deep anesthesia were homogenized in phenol (Omega Bio-Tek Inc., Norcross, GA, USA) and stored at -80°C.

Total RNA in hypothalamus samples was extracted using phenol and reverse transcribed by RT-PCR using One-Step SYBR RT-PCR Kits (Takara Bio Inc., Shiga, Japan) with specific primer pairs designed using Primer 3 Plus software (**Table 2**) and a Piko Real PCR system (Thermo Fisher Scientific Inc., Waltham, MA, USA). The relative expression of target genes was normalized to that of *Gapdh*. The data were analyzed using the ΔΔCt method. Melt curves were analyzed to identify non-specific products.

**Table 2:** Primers.

	Forward	Reverse
*Gapdh*	TGGTGAAGGTCGGTGTGAAC	AATGAAGGGGTCGTTGATGG
*Npy*	ACCCTCGCTCTATCTCTGCTC	TATCTGGCCATGTCCTCTGC
*Pomc*	GATGTGTGGAGCTGGTG	GGCTGTTCATCTCCGTTG
*Cart*	CTGGACATCTACTCTGCCGTGG	GTTCCTCGGGGACAGTCACACAGC

### 2.11 Experimental details

Experiment 1: Male ICR mice were assigned to groups fed with MF, HFD-cellulose, or HFD-RMD (n = 6−8), given access to tap water *ad libitum* and kept in individual cages for 10 weeks. The body weight and food intake were measured weekly. Subsequently, we prepared other ICR male mice and fed them with either HFD-cellulose or HFD-RMD (n = 7) in individual cages for 8 weeks. We collected fresh feces at ZT10 before the experiment starting and after the kept each condition for 8 weeks, to analyze the gut microbiota composition. Moreover, we also collected cecal contents at ZT10 after the kept each condition for 8 weeks to analyze the SCFAs content.

**Figure 1:**
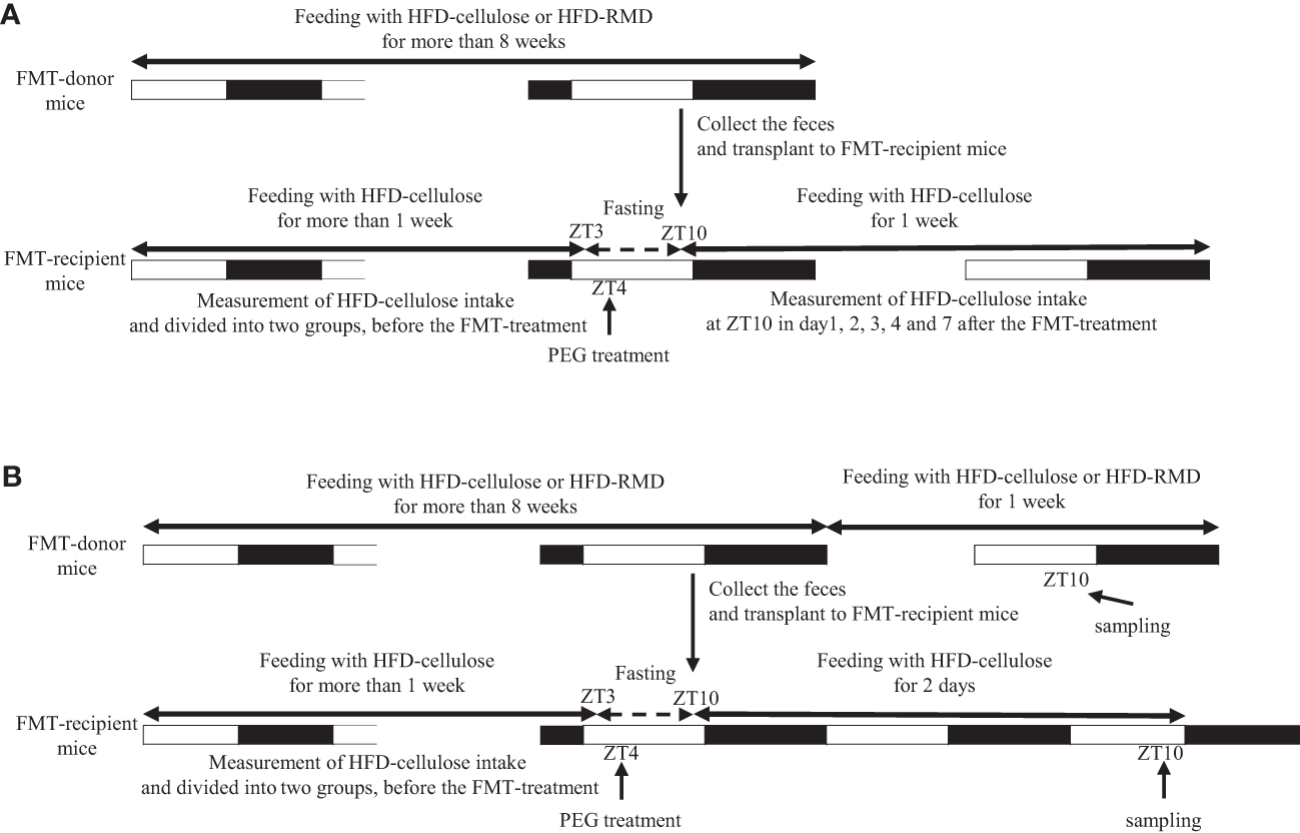
Protocols of fecal microbiota transplantation in experiments 2 and 3. Experiments **(A)** 2 and **(B)** 3.

Experiment 2: Male ICR mice were assigned to groups fed with an HFD-cellulose or HFD-RMD for > 8 weeks as the FMT-donor mice (HFD-cellulose and HFD-RMD groups; n = 8). We also assigned other ICR male mice (n = 7−8) as the FMT-recipient mice (FMT-HFD-cellulose and FMT-HFD-RMD groups) and fed them with an HFD-cellulose for one week of habituation before undergoing the FMT. Thereafter, the FMT-recipient mice were fed with an HFD-cellulose. We measured the volume of HFD-cellulose at ZT10 on days 1−4 and 7 to calculate the daily intake (**Figure 1A**).

Experiment 3: We fed ICR male mice with an HFD-cellulose or HFD-RMD groups for > 8 weeks (HFD-cellulose and HFD-RMD groups; n = 4). We also prepared ICR male mice (n = 7−8) as the FMT-recipient mice (FMT-HFD-cellulose and FMT-HFD-RMD groups) and fed them with an HFD-cellulose for one week of habituation before undergoing the FMT. Serum, hypothalamus, cecal content, and feces of the FMT-recipient mice were collected at ZT10 two days after the FMT. Moreover, serum, hypothalamus, and cecal contents of FMT-donor mice were also collected at ZT10 one week after the feces collection (**Figure 1B**). Levels of gut hormones associated with appetite regulation that are produced in the gut were measured in serum, appetite-related neuropeptide mRNA expression was assessed in the hypothalamus, SCFAs in the cecal content were quantified, and the gut microbiota content was analyzed using feces. We collected samples from FMT-recipient mice two days after FMT because HFD-cellulose intake was stably suppressed in the FMT-HFD-RMD group at that time. Additionally, we collected samples from FMT-donor mice one week after feces collection to allow them to recover from the stress of feces collection.

In this experiment, we collected samples at ZT10, and we have mainly two reasons. First, feces were collected more easily. Indeed, a previous study in mice showed that fecal output showed a circadian rhythm and that fecal output during the inactive period was significantly lower than that during the active period (Hoogerwerf et al., 2010). Therefore, at approximately ZT10, just before the active period, we expected that mice would retain more feces in the colon. Second, we expected that each parameter at approximately ZT10 would reflect feeding behavior during the subsequent active period. Indeed, a previous study has shown that mice predominantly consume food during the active period (Chaix et al., 2019). Therefore, we expected that sample collection at ZT10 would be appropriate.

### 2.12 Statistical analysis

All values are expressed as means ± standard error of the mean (SEM). All data were statistically analyzed using GraphPad Prism v. 6.03 (GraphPad Software, San Diego, CA, USA). Normal or non-normal data distribution was determined by assessing equal variation using D’Agostino–Pearson normality tests, Kolmogorov-Smirnov t tests, and one-sample t-tests. Bias was assessed using F-value and Bartlett test. Significance between two independent groups was assessed by parametric analysis using unpaired t-tests, and abnormally distributed data were assessed by non-parametric analysis using Mann-Whitney tests and by Kruskal-Wallis tests with Dunn multiple comparison tests. Two factors requiring non-parametric analysis were assessed using Mann Whitney tests with false discovery rate multiple testing correction.

## 3 Results

### 3.1 Effects of RMD on body weight, food intake volume, gut microbiota composition, and SCFAs in the cecal contents

**Figure 2:**
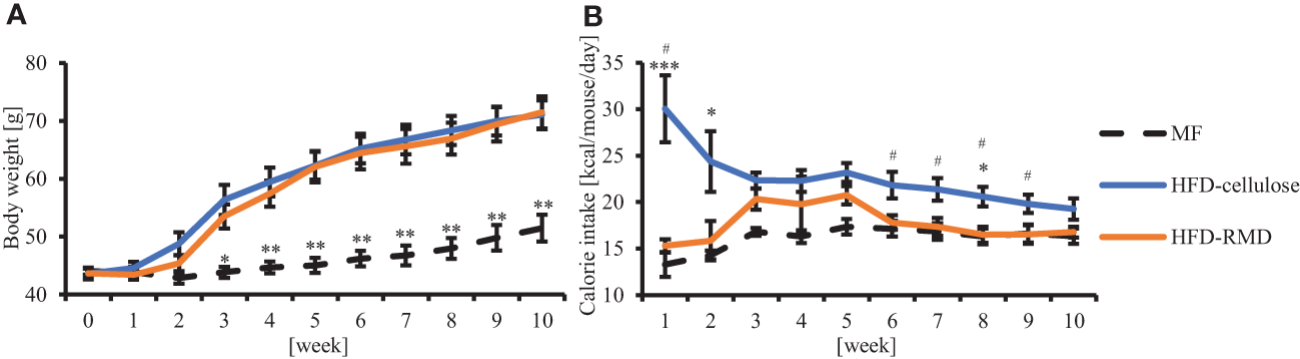
Effects of resistant maltodextrin (RMD) on body weight and caloric intake. Changes in average **(A)** body weight and **(B)** caloric intake. (MF, n = 7; HFD-celllose, n = 8; HFD-RMD, n = 6). Data are shown as means ± standard error of the mean (SEM). * p < 0.05, ** p < 0.01, *** p < 0.001 vs the HFD-RMD group (Kruskal-Wallis test with Dunn’s multiple comparisons test). # p < 0.05 vs the HFD-RMD group (Mann Whitney test).

We investigated the effects of water-soluble or -insoluble dietary fiber on food intake and body weight. We fed mice with MF, HFD-cellulose, or HFD-RMD for 10 weeks, monitored their body weight and food volume weekly, and calculated their food intake. Body weight changes in the HFD-RMD group were similar to those in the HFD-cellulose group and were significantly higher than those in the MF group (**Figure 2A**). Caloric intake in the HFD-RMD group was significantly lower than that in the HFD-cellulose group after the beginning of feeding and at week 6−9 (**Figure 2B**).

**Figure 3:**
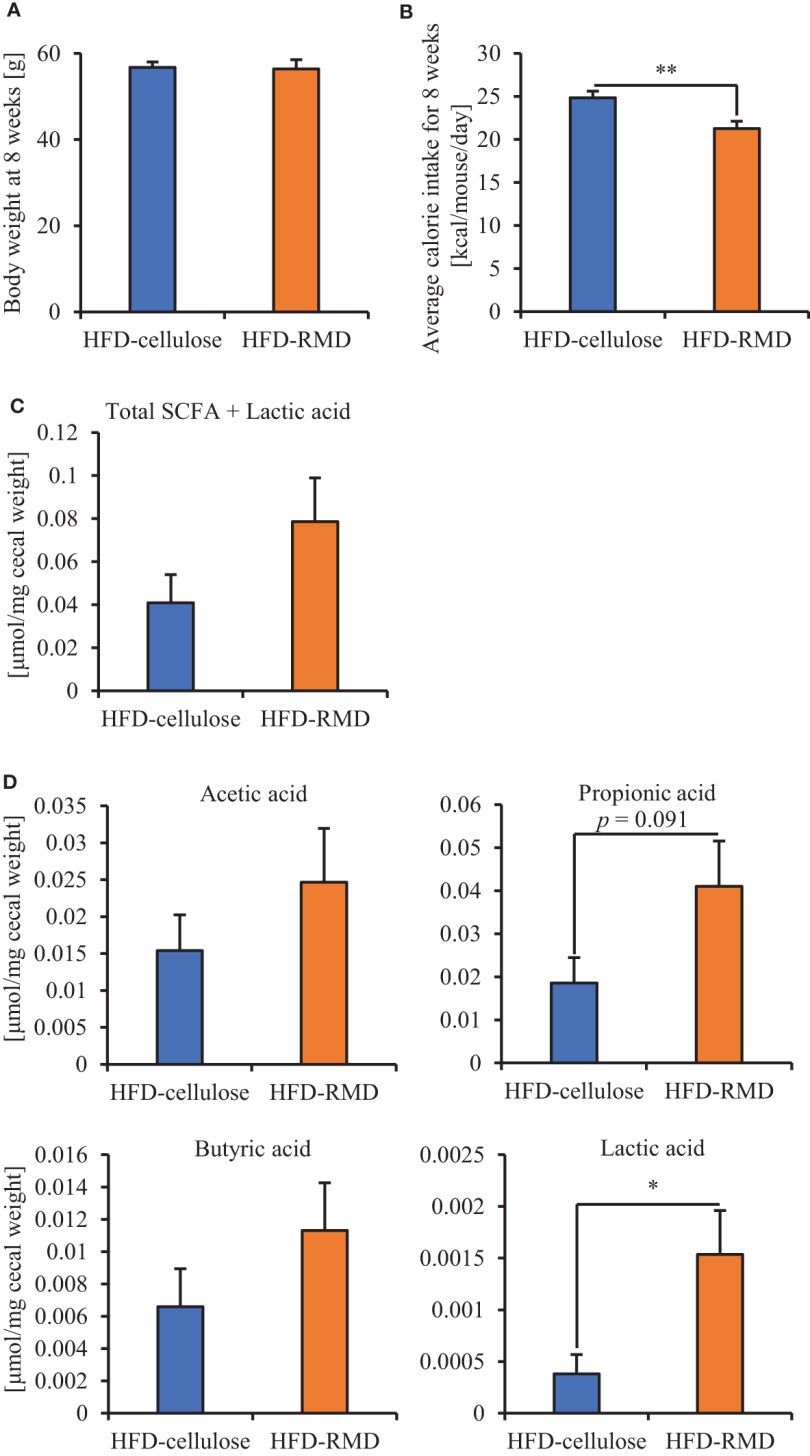
Effects of RMD on short-chain fatty acids (SCFAs). **(A, B)** Body weight at week 8 and average caloric intake for 8 weeks. **(C, D)** The SCFAs components and lactic acid were measured from cecal contents (n = 6). We failed to extract SCFAs one mouse in each group. Data are presented as the mean ± SEM. * p < 0.05, ** p < 0.01 (unpaired t-test).

**Figure 4:**
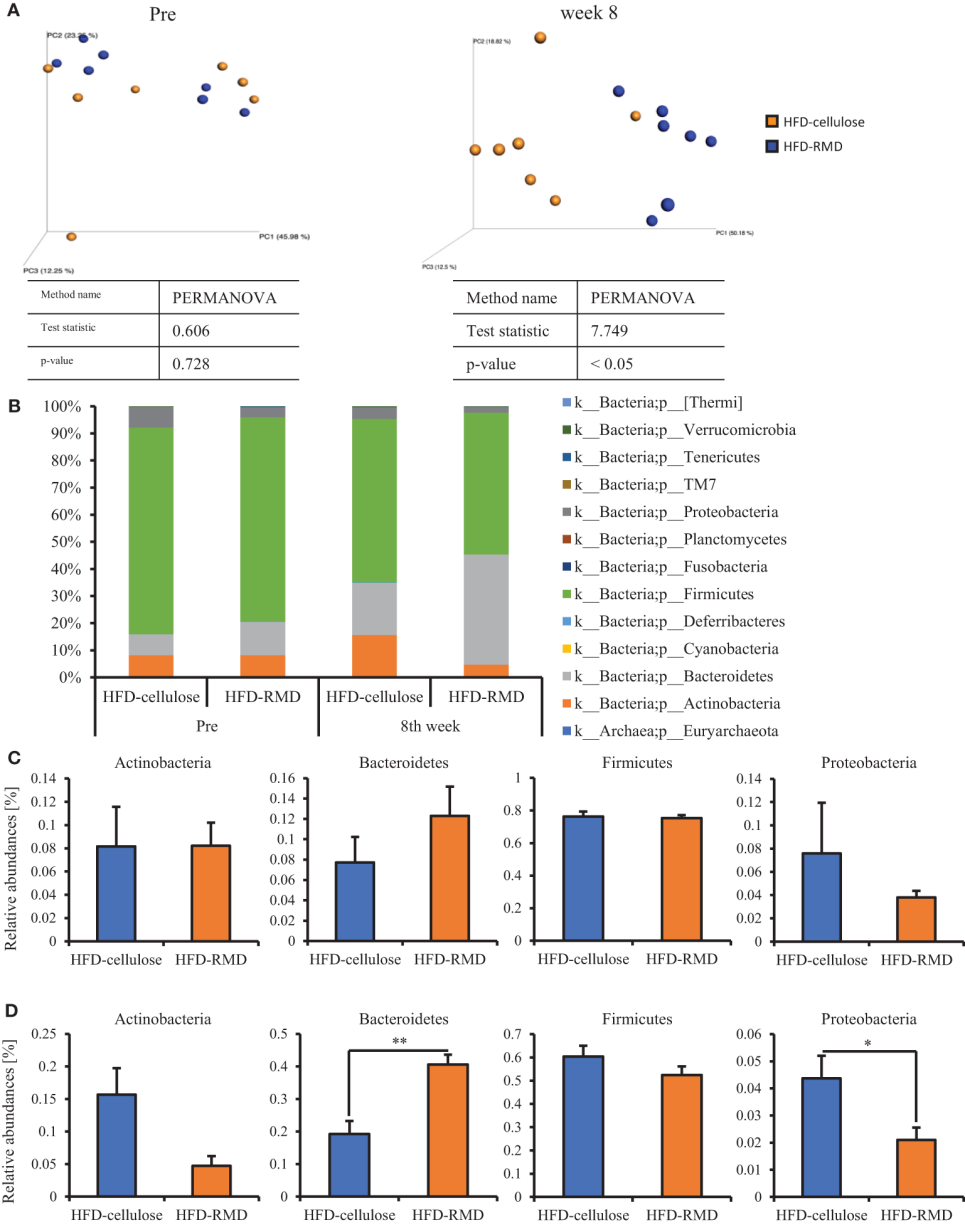
Effects of HFD-R on gut microbiota and relative abundance of some bacteria. **(A)** UniFrac principal coordinate analysis (PCoA) weighted intestinal microbiota. **(B)** Composition the intestinal microbiota. Relative abundance at phylum level **(C)** before and **(D)** after mice were fed with each diet for 8 weeks. All data are shown as means ± SEM (n = 7). * p < 0.05, ** p < 0.01 (unpaired t-test). genus level (*Bacteroides*, *Parabacteroides*, and *Dorea*) in mice maintained under each diet for 8 weeks.

**Figure 5:**
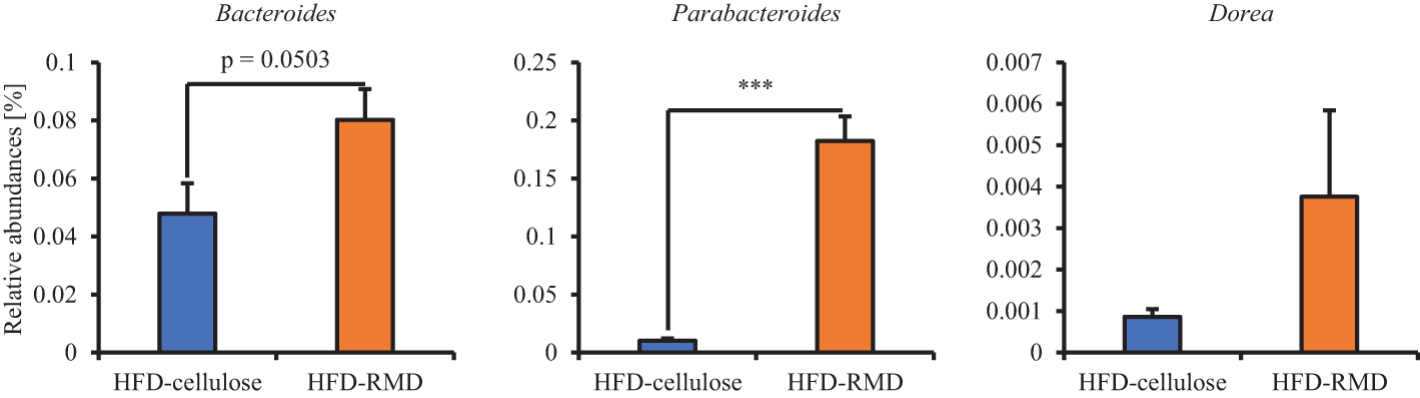
Effects of resistant maltodextrin on relative abundance of some bacteria. Relative abundance at All data are shown as means ± SEM (n = 7). *** p < 0.001 (Mann Whitney tests).

Subsequently, we prepared HFD-cellulose and HFD-RMD groups using other mice. We collected feces from mice before and 8 weeks after starting the HFD-cellulose or the HFD-RMD groups. We also collected cecal contents at week 8 because differences in caloric intake were significant in both groups. Body weights in both groups were similar after being maintained under each condition for eight weeks (**Figure 3A**). Moreover, the average calorie intake over 8 weeks in the HFD-RMD group was significantly lower than that in the HFD-cellulose group (**Figure 3B**). These results were similar to the above results (**Figure 2**). Propionic acid levels in the HFD-RMD group tended to be higher than those in the HFD-cellulose group (**Figure 3D**). The lactic acid levels in the HFD-RMD group were significantly higher than those in the HFD-cellulose group (**Figure 3D**). The total SCFAs, acetic acid, and butyric acid levels in the HFD-RMD group showed similar levels as those in the HFD-cellulose group (**Figures 3C, D**). **Figure 4** shows the gut microbiota composition in feces samples. Weighted UniFrac PCoA plots of the gut microbiota composition revealed a similar composition in both groups before starting the experiment, and significant differences after feeding with an HFD-RMD or HFD-cellulose for 8 weeks (**Figure 4A**). The gut microbiota composition and relative abundance of *Actinobacteria*, *Bacteroidetes*, *Firmicutes*, and *Proteobacteria* at the phylum level were similar between the HFD-cellulose and HFD-RMD groups before starting the experiment (**Figures 4B, C**). However, after being maintained under each condition for 8 weeks, the gut microbiota composition differed between the HFD-cellulose and HFD-RMD groups (**Figure 4B**). The relative abundance of *Bacteroidetes* and *Proteobacteria* in the gut microbiota significantly differed between both groups (**Figure 4D**). The abundances of *Actinobacteria* and *Firmicutes* showed similar levels in both groups (**Figure 4D**). Subsequently, we analyzed some bacteria at the genus level. The relative abundance of *Bacteroides*, which produces acetic acids, in the HFD-RMD group tended to be higher than that in the HFD-cellulose group, and the relative abundance of *Parabacteroides*, which produces acetic acids, in the HFD-RMD group was significantly higher than that in the HFD-cellulose group (**Figure 5**). The relative abundance of *Dorea*, which produces butyrate, showed similar levels in both groups (**Figure 5**) (Martín-Núñez et al., 2021; Namted et al., 2022).

These results suggested that acute and long-term feeding with RMD feeding inhibited the increased calorie intake with HFD and changed the gut microbiota composition compared with cellulose feeding.

### 3.2 Effects of the gut microbiota composition on HFD intake

**Figure 6:**
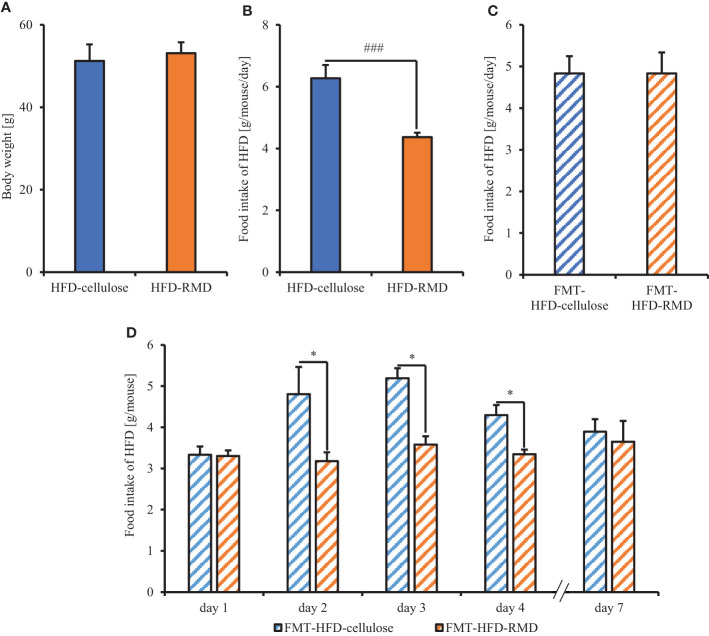
Food intake of mice transplanted with fecal microbiota from mice fed with HFD supplemented with or without RMD. **(A, B)** Body weight and average each food intake level in the FMT-donor mice before the FMT-treatment. We kept both groups under each feeding condition for more than 8 weeks and measured the food intake 1 week before the FMT. **(C)** Average HFD-cellulose intake levels in the FMT-recipient mice before the FMT-treatment for 2 days before the FMT. **(D)** Change in average food intake of HFD-cellulose during each day. Data are presented as the mean ± SEM (the HFD-cellulose and HFD-RMD groups, n = 8; the FMT-HFD-cellulose group, n = 7; the FMT-HFD-RMD group, n = 8). * p < 0.05 (Mann Whitney test with false discovery rate multiple testing correction). ### p < 0.001 (Mann Whitney test).

We showed that RMD feeding changed gut microbiota composition and suppressed the increased calorie intake with HFD. Therefore, we investigated the effects of the gut microbiota composition on the increased calorie intake with HFD in the FMT-recipient mice (FMT-HFD-cellulose and FMT-HFD-RMD groups) transplanted with feces collected from the FMT-donor mice (HFD-cellulose and HFD-RMD groups). Before the FMT, we measured body weight and food intake in the FMT-donor mice and measured HFD-cellulose intake in the FMT-recipient mice. After the FMT, the FMT-HFD-cellulose and FMT-HFD-RMD groups were fed with an HFD-cellulose, and the intake volumes were monitored (**Figure 1A**). Before FMT in the FMT-donor mice, the body weight in the HFD-RMD group was similar to that in the HFD-cellulose group (**Figure 6A**), and food intake in the HFD-RMD group was significantly lower than that in the HFD-cellulose group (**Figure 6B**). Before FMT in the FMT-recipient mice, HFD-cellulose intake was similar in both groups (**Figure 6C**). Intake of HFD-cellulose was significantly lower in the FMT-HFD-RMD group than that in the FMT-HFD-cellulose group at days 2−4 after the FMT (**Figure 6D**). This effect disappeared one week after the FMT (**Figure 6D**). Overall, these results suggested that long-term RMD feeding would inhibit excessive calorie intake in HFD-cellulose in the HFD-RMD group by changing the gut microbiota composition.

### 3.3 Cecal SCFAs levels, serum levels of appetite-associated gut hormones, and gene transcripts of appetite-associated neuropeptides in hypothalamus from the FMT-donor and recipient mice

The above findings indicated that long-term RMD feeding altered the gut microbiota composition, and caused the suppression of excessive calorie intake in HFD-cellulose. We then investigated the mechanism of this suppression effect, in the FMT-donor and recipient mice under the same conditions as Experiment 2. We collected samples from the FMT-recipient mice two days after the FMT. Moreover, we collected samples from FMT-donor mice at one week after the FMT (**Figure 1B**).

**Figure 7:**
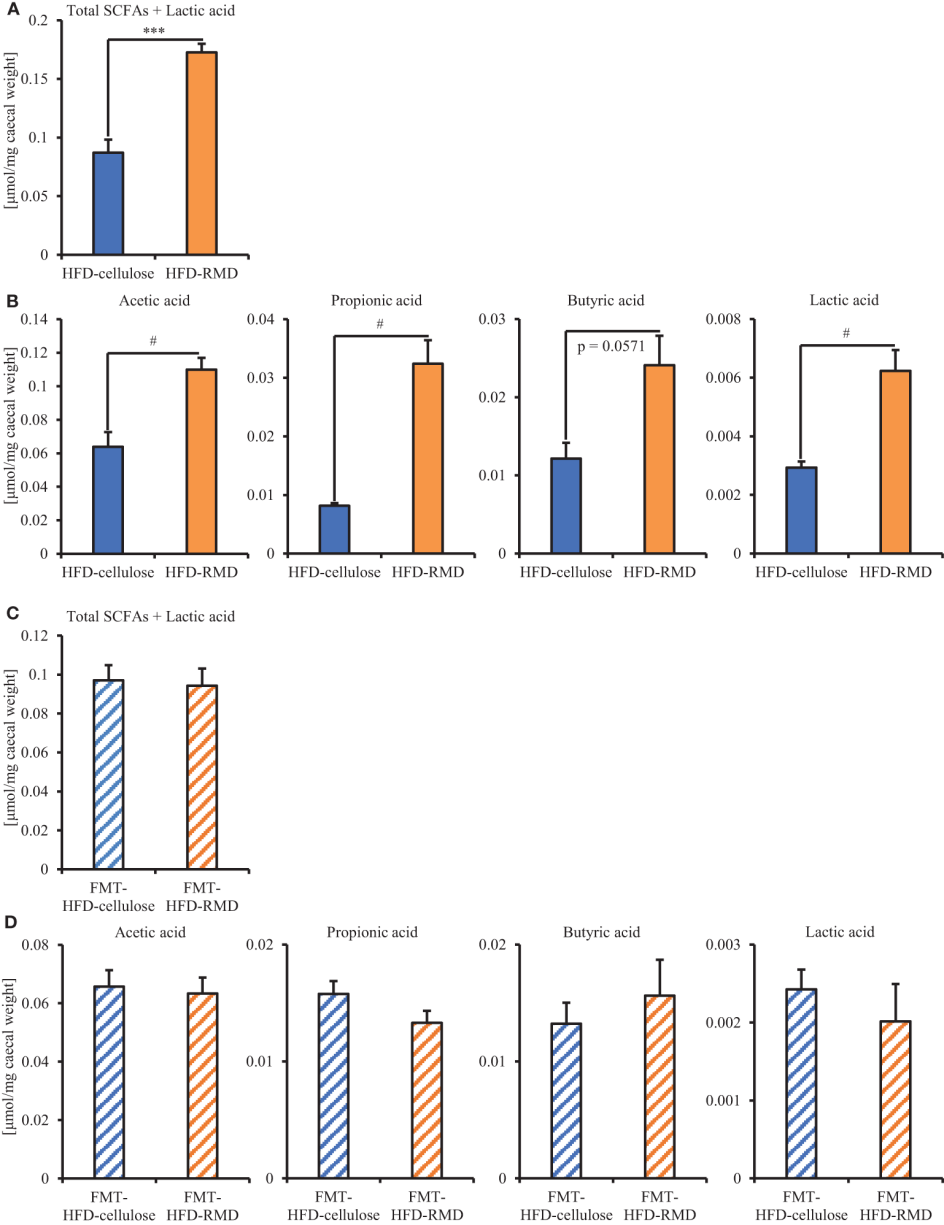
Quantitation of SCFAs and lactic acid in FMT donor and recipient mice. Total SCFAs, each SCFAs components, and lactic acid quantified in cecal contents from **(A, B)** FMT-donor and **(C, D)** FMT-recipient mice. Data are shown as means ± SEM (donors, n = 4; recipients, n = 7). *** p < 0.001 (unpaired t-test). # p < 0.05 (Mann Whitney test).

**Figure 8:**
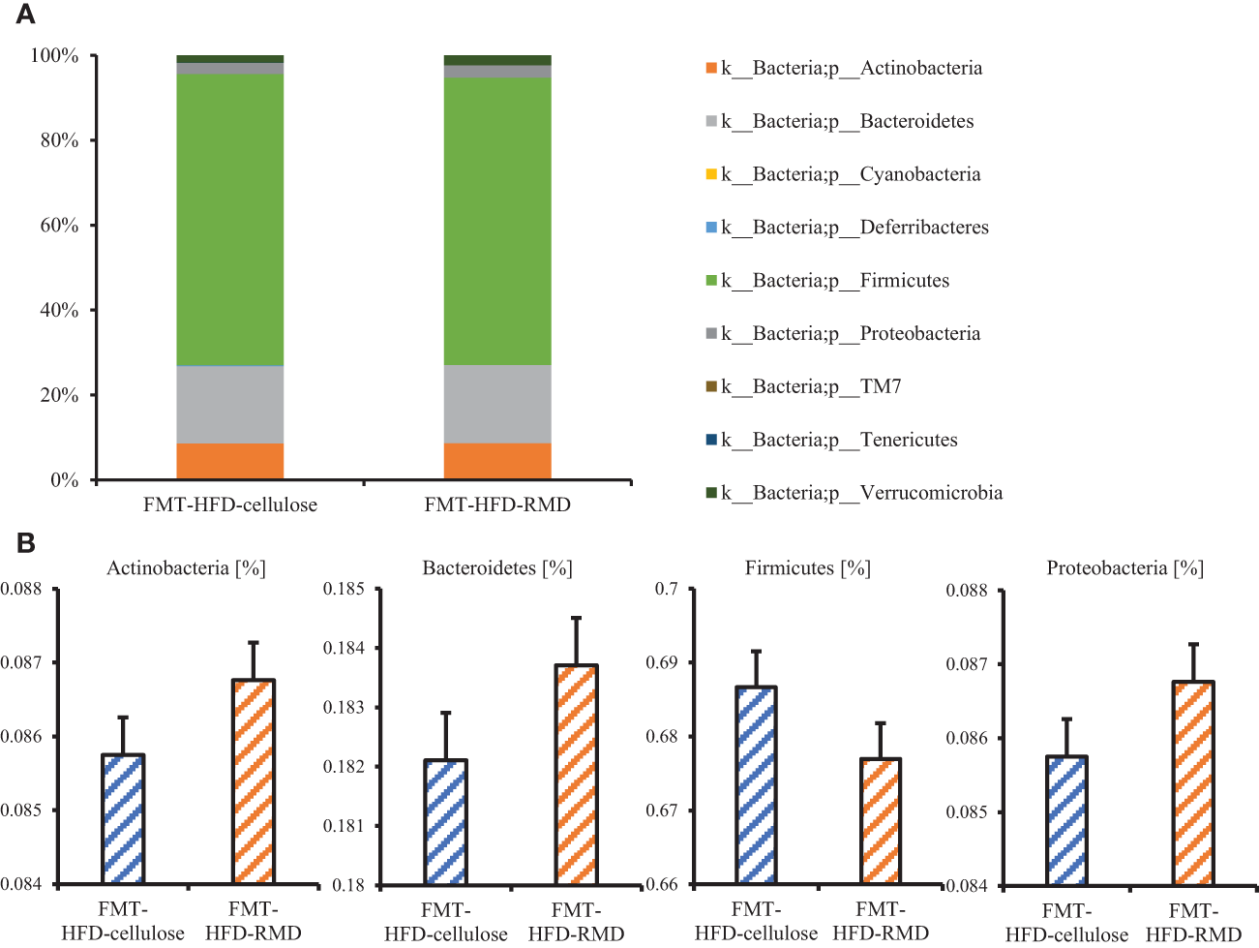
The gut microbiota component in the FMT-HFD-cellulose and -FMT-HFD-RMD groups. **(A)** Gut microbiota composition and **(B)** relative bacterial abundance at phylum level in FMT-HFD-cellulose and FMT-HFD-RMD groups 2 days after the FMT. All data are shown as means ± SEM (n = 7).

The results showed that the consumption of HFD-RMD significantly increased total SCFAs and individual SCFAs levels in the cecal contents of the FMT-donor mice (the HFD-RMD group), compared with the HFD-cellulose group (**Figures 7A, B**). In contrast, total SCFAs and individual SCFAs levels were similar between both groups of the FMT-recipient mice (the FMT-HFD-cellulose and FMT-HFD-RMD groups) (**Figures 7C, D**). In addition, we analyzed the gut microbiota composition in feces from the FMT-HFD-cellulose and FMT-HFD-RMD groups (**Figure 8**). At the phylum level, gut microbiota composition was similar between the FMT-HFD-cellulose and FMT-HFD-RMD groups (**Figure 8A**). Moreover, the relative abundances of *Actinobacteria*, *Bacteroidetes*, *Firmicutes*, and *Proteobacteria* showed similar levels in both groups (**Figure 8B**).

**Figure 9:**
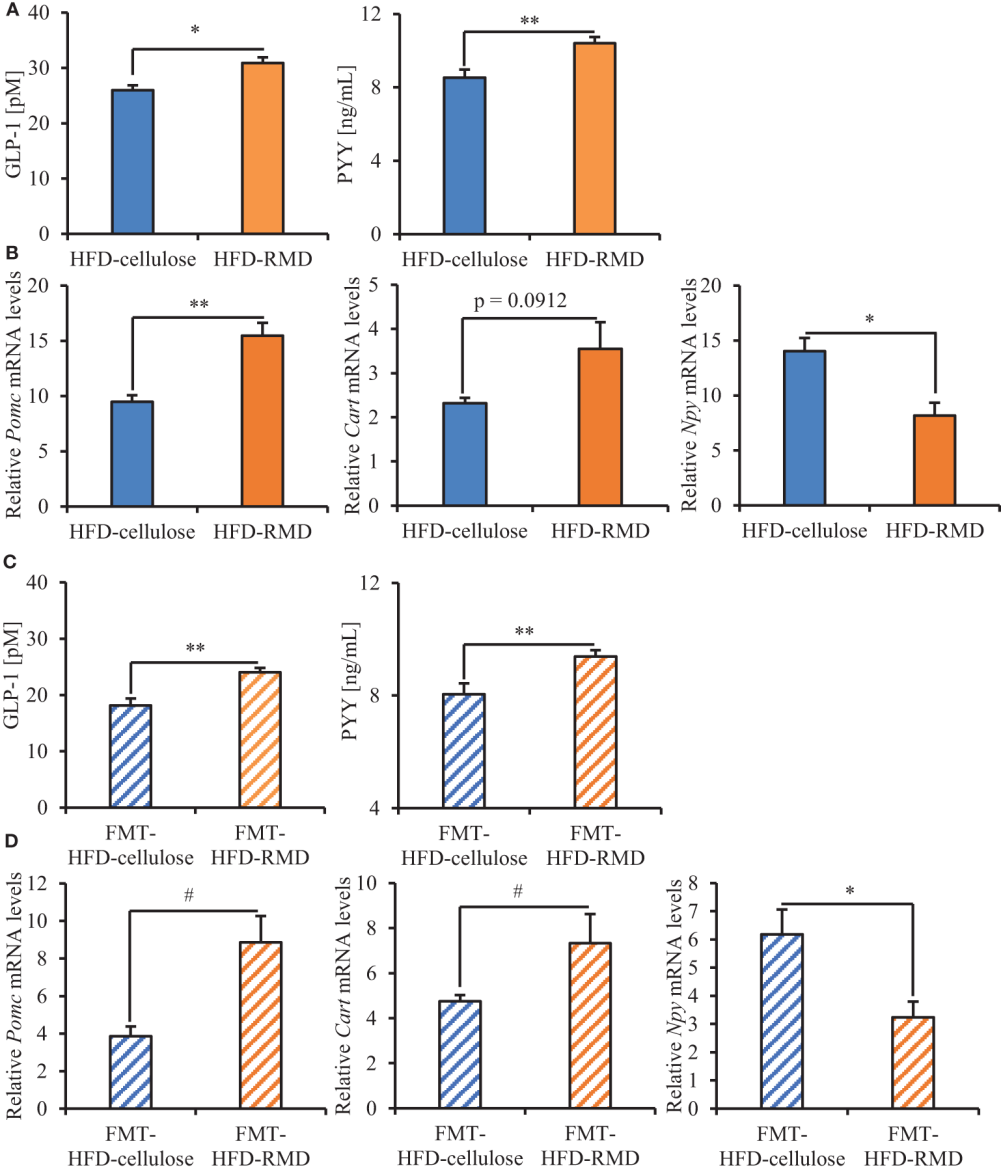
Serum levels of appetite-related gut hormones and neuropeptides mRNA in hypothalamus of the FMT-donor and recipient mice. Serum values for active GLP-1 and PYY in **(A)** the FMT-donor and **(C)** recipient mice. **(B)** Hypothalamic levels of *Pomc*, *Cart*, and *Npy* mRNA in **(B)** the FMT-donor and **(D)** recipient mice. Data are shown as means ± SEM (the FMT-donor mice, n = 4; FMT-recipient mice, n = 7). * p < 0.05, ** p < 0.01 (unpaired t-test). # p < 0.05 (Mann Whitney test).

We analyzed serum levels of appetite-related gut hormones (active GLP-1 and PYY) that stimulate satiety. Levels of active GLP-1 and of PYY were significantly higher in the HFD-RMD group than the HFD-cellulose group of the FMT-donor mice (**Figure 9A**). The results were notably similar between the FMT-donor and recipient mice (**Figures 9A, C**). Levels of serum active GLP-1 and of PYY were significantly higher in the FMT-HFD-RMD group than the FMT-HFD-cellulose group of the FMT-recipient mice (**Figure 9C**).

We analyzed the transcriptional levels of the appetite-related neuropeptides POMC and CART (appetite suppression), as well as NPY (appetite stimulation) in the hypothalamus (Chen et al., 2007; Hill, 2010). Significantly less *Npy* was transcribed in the HFD-RMD group than HFD-cellulose group of the FMT-donor mice. Levels of *Pomc* and *Cart* transcription were significantly higher and higher, respectively, in the HFD-RMD group than in the HFD-cellulose group (**Figure 9B**). The results of the FMT-recipient and donor mice were notably similar (**Figures 9B, D**). Significantly more *Pomc* and *Cart*, and significantly less *Npy* were transcribed in the FMT-HFD-RMD group than in the FMT-HFD-cellulose group of the FMT-recipient mice (**Figure 9D**).

Serum appetite-related gut hormones (satiety stimulators) were increased, appetite-related neuropeptide gene transcription was changed in the hypothalamus, and excessive calorie intake in HFD-cellulose was inhibited in the HFD-RMD and FMT-HFD-RMD groups. Levels of SCFAs in the cecal contents and the gut microbiota composition were similar in the FMT-recipient mice (FMT-HFD-cellulose and FMT-HFD-RMD groups), suggesting that the diet immediately affected SCFAs and gut microbiota composition. Our findings indicated that the FMT-HFD-RMD group would maintain the gut macrobacteria, which were increased by long-term RMD feeding and transplanted from the HFD-RMD group, despite feeding this group with HFD-cellulose.

## 4 Discussion

The present study found that feeding mice with RMD for long and short terms inhibited the increased calorie intake with HFD and that long-term RMD changed the gut microbiota composition and increased the amount of cecal SCFAs levels. Furthermore, the gut microbiota composition, which was changed by long-term RMD feeding, increased serum active GLP-1 and PYY levels and altered the expression of appetite-related neuropeptide genes in the hypothalamus. We also found that RMD even suppressed excessive calorie intake in HFD-cellulose by the FMT-recipient mice transplanted with gut microbiota from mice fed with RMD, even when the FMT-recipient mice were fed only with an HFD-cellulose. Moreover, the FMT-recipient mice, transplanted with gut microbiota from mice fed with RMD, showed the increases of serum active GLP-1 and PYY levels and the changes of the expression of appetite-related neuropeptide genes in the hypothalamus. The present findings indicate that the increased calorie intake with HFD would be suppressed through changes in the gut microbiota composition caused by long-term RMD feeding, which increased the amount of SCFAs, as well as active GLP-1 and PYY secretion, and affected appetite-related neuropeptide gene transcription in the hypothalamus.

We found that long-term RMD feeding inhibited the increased calorie intake with HFD. One week of RMD feeding decreases HFD intake and contributes to a decrease in postprandial hunger and the maintenance of satiety in rats and humans, respectively (Ye et al., 2015; Hira et al., 2018). However, previous studies of rats found that long-term RMD feeding (~ 8 weeks) did not affect NFD and HFD intake (Hira et al., 2015; Hira et al., 2018). We have no correct idea why our findings and previous studies’ results were differences. One of differences between previous study and our study is strain (rat or mouse). These results indicated that acute and long-term RMD feeding inhibited the increased calorie intake with HFD in mice.

We revealed that long-term RMD supplementation changed the gut microbiota composition and increased the amount of cecal SCFAs. Previous studies have suggested that RMD is a prebiotic fiber that modifies the gut microbiota composition in humans and leads to increased SCFAs production (Fastinger et al., 2008; Hashizume et al., 2012). Moreover, fructooligosaccharides, burdock (*Arctium lappa*) root, and other water-soluble dietary fibers exert similar effects in rodents (Hira et al., 2018; Watanabe et al., 2020). These results suggest that the intake of water-soluble dietary fibers, including RMD, would modify the gut microbiota composition, leading to increase of cecal SCFAs production.

We found that dietary RMD supplementation increased serum levels of active GLP-1 and of PYY and that these levels were also increased in mice transplanted with gut microbiota from the HFD-RMD group. Both propionic and butyric acid increase *Pyy* transcription and PYY secretion in NCI-h716 enteroendocrine cells that model human GLP-1 secreting scarce *in vitro* (Larraufie et al., 2018). The isolated perfused rat colon stimulated by acetic or butyric acid increases GLP-1 secretion (Christiansen et al., 2018). GPR 41 or 43 activation is involved in the increase in GLP-1 secretion induced by SCFAs (Blad et al., 2012). In addition, feeding rats with the RMD for various duration increases serum GLP-1 and GLP-1 levels in the rat cecum (Hira et al., 2015; Hira et al., 2018). Dietary supplementation with RMD for one week increases postprandial satisfaction as well as plasma GLP-1 and PYY levels in humans (Hira et al., 2015; Ye et al., 2015; Hira et al., 2018). However, whether RMD directly or indirectly increases GLP-1 and PYY secretion levels remained unclear. Taken together, our results indicated that the RMD feeding indirectly would increase active GLP-1 and PYY secretion levels by changing the gut microbiota composition, which leads to the suppression of the increased calorie intake with HFD.

We revealed that the RMD supplementation significantly increased and decreased *Pomc* (appetite suppression-related neuropeptide gene) and *Npy* (appetite stimulation-related neuropeptide gene) transcription in the hypothalamus. We also found that in the hypothalamus, mice transplanted with gut microbiota from the HFD-RMD group showed significantly increased *Pomc* and *Cart* transcription levels and significantly decreased *Npy* transcription level. Previous studies reported that administration of GLP-1 or PYY to fasted rats suppressed food intake after fasting and significantly increased and decreased the *Pomc* and *Npy* transcriptional levels in the hypothalamus, respectively (Challis et al., 2003; Seo et al., 2008). These results suggested that the increased serum GLP-1 and PYY levels would affect *Pomc*, *Cart*, and *Npy* transcription in the hypothalamus, which in turn would suppress the increased calorie intake with HFD.

In the FMT experiment, we revealed that the FMT-HFD-RMD group showed similar HFD-cellulose intake throughout the week after FMT and that the FMT-HFD-cellulose group showed an increase in HFD-cellulose intake in days 2–4 compared to days 1 and 7 after FMT. Indeed, a previous study in rats indicated that re-feeding with HFD increased calorie intake from the first or second day after fasting compared with re-feeding with NFD, and that calorie intake from HFD changed to the same level as caloric intake from NFD approximately one week after re-feeding (Moriya et al., 2018). These results suggest that changes in gut microbiota composition altered by RMD supplementation would suppress the increase in excessive calorie intake in HFD a few days after re-feeding with HFD.

The intake of HFD-cellulose on day 7 after the FMT was similar between the FMT-HFD-cellulose and FMT-HFD-RMD groups. Moreover, the FMT-HFD-cellulose and FMT-HFD-RMD groups had similar SCFAs levels and gut microbiota composition on day 2 after the FMT. Previous studies on probiotics conducted administration with probiotics at various intervals, from once a day to once a week (Chen et al., 2005; Wrzosek et al., 2018; Foroozan et al., 2021). These results suggested that probiotics effects would remain for several days, which would vary depending on the gut bacteria, food, and other conditions.

We also observed that the FMT-HFD-cellulose and FMT-HFD-RMD groups showed similar SCFAs levels and relative abundance of the gut microbiota components. The reasons would be that we fed the FMT-recipient mice (the FMT-HFD-cellulose and FMT-HFD-RMD groups) with same food including only water-insoluble dietary fiber (cellulose) after the FMT. Indeed, previous studies have shown that water-insoluble dietary fiber decreases the production of SCFAs (Tahara et al., 2018), and that the gut microbiota composition changes rapidly after dietary ingestion and responds rapidly to the altered diet (Zhou et al., 2023). Therefore, we hypothesized that the gut bacteria induced by the RMD feeding would be present in the intestinal of the FMT-HFD-RMD group until a few days after the FMT. Indeed, we observed the effects of RMD on the increased calorie intake with HFD, serum appetite-related gut hormones levels, and appetite-related neuropeptides genes transcription levels in the FMT-HFD-RMD group. These results suggest that the gut bacteria, which would be increased by the RMD feeding and involved in suppressing the increased calorie intake with HFD, may have been present in the intestines of FMT-recipient mice for a few days.

We found that after only one FMT, the FMT-HFD-RMD group showed lower calorie intake with HFD-cellulose than the FMT-HFD-cellulose group at days 2–4 after FMT. A previous study performed FMT at various intervals (once to twice a week) and suggested that FMT once a week appeared to be the best compromise, as it allowed the engraftment of *Faecalibacterium* and a higher diversity of bacteria belonging to the order Bacteroidales (Wrzosek et al., 2018). These results indicate that repeated weekly FMT for several weeks may continuously inhibit the increased calorie intake associated with HFD.

We used HFD-RMD, containing 4% RMD, in this study. Previous studies also evaluated the effects of an HFD supplemented with 4−5% RMD (Hira et al., 2015; Hira et al., 2018), which in rodents study is equivalent to 20–30 g/day in humans (Parnell and Reimer, 2012; Cluny et al., 2015) that complies with the recommended levels of dietary fiber (Turner and Lupton, 2011). Moreover, RMD (continuous intake of 60 g/day for three months or acute intake of 50 g) has never been reported to induce severe gastrointestinal symptoms or diarrhea (Nomura et al., 1992; Okuma and Matsuda, 2002). Therefore, we presumed that 4% RMD supplementation would be suitable for the present study.

This study had six limitations. First, we kept mice in the individual cages in all experiments, because we needed to monitor individual food intake for various term. However, it is said that single housing would be stressful for animals. We were trying to reduce the effects of stress by keeping mice in the HFD-cellulose and HFD-RMD groups under single housing for more than eight weeks before the FMT (Experiment 1-3), and by keeping FMT-recipient mice (the FMT-HFD-cellulose and FMT-HFD-RMD groups) under single housing for one week before the FMT (Experiment 2 and 3). Indeed, previous study in mice showed that food intake level in single housed group was similar to that in pair housed group (Buckinx et al., 2021). Second, we did not identify which gut bacteria were increased by RMD feeding and directly involved in the increased secretion of GLP-1 and PYY. However, we found that serum active GLP-1 and PYY were increased in the FMT-HFD-RMD group and that the FMT-HFD-RMD group showed significantly lower HFD-cellulose intake than the HFD-RMD group. Thus, we predicted that the intestines of FMT-HFD-RMD group (FMT-recipient mice) would have gut bacteria that were increased by RMD feeding and involved in increasing the serum GLP-1 and PYY levels. Third, we did not use germ-free (GF) mice as FMT-recipient mice in experiments 2 and 3. Although PEG can reduce ~ 90% of the total gut bacteria in the luminal and mucosal intestinal microbiota (Wrzosek et al., 2018), it does not eliminate all gut bacteria. We could not address this issue using GF mice due to space constraints at our institution. Fourth, there are no studies on the effect of RMD on fat preference in animals; thus, we were unable to analyze these effects. However, our results in mice and previous studies in rats have shown that RMD consumption increases the secretion of appetite-related gut hormones, such as GLP-1 and PYY (Hira et al., 2015; Ye et al., 2015; Hira et al., 2018). Therefore, we hypothesized that the effect of RMD on fat preference is limited. Fifth, we did not confirm the effects of RMD on body weight gain. We had no data on the amount of fat after feeding RMD for 8 weeks, energy expenditure in the whole body, brown adipose tissue activity, energy excretion in the feces, or the weight of fecal output. Future studies are required to verify the effects of RMD on body weight gain. Finally, in FMT, we treated the fecal matter as quickly as possible during preparation, but we did not use precautions to limit the oxygenation of fecal slurries. Therefore, in the future, experiments may be required to use precaution to limit the oxygenation of fecal slurries.

**Figure 10:**
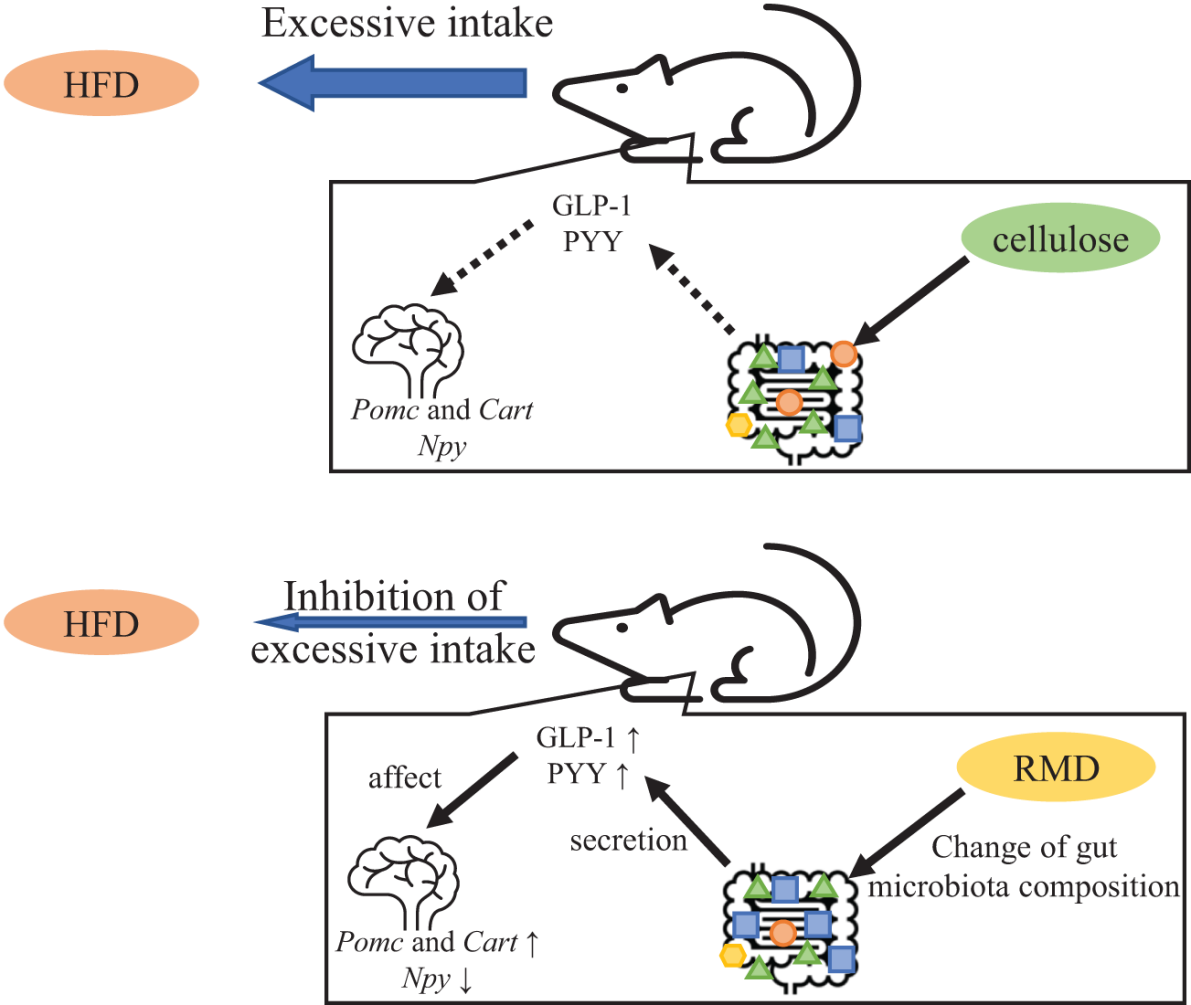
Predicted mechanism of appetite suppression and satiety.

In conclusion, our findings provide evidence that long-term RMD feeding suppresses excessive calorie intake in mice even when fed with a highly palatable HFD that induces such intake. We also showed that these effects are mediated by changes in the gut microbiota components, increases in serum active GLP-1 and PYY levels, and changes in appetite-related neuropeptide gene transcription (**Figure 10**). These results suggested that RMD supplementation contributes to appetite suppression without stress and might represent a new therapeutic strategy for obese patients with excessive caloric intake. However, it is not yet known whether the results of our study can be completely extrapolated to humans. Therefore, additional experiments would be required to verify these possibilities.

## Data availability statement

The datasets presented in this study can be found in online repositories. The data presented in the study are deposited in the DDBJ Sequence Read Archive (DRA), accession number DRA015845. The names of the repository/repositories and accession number(s) can be found below: https://ddbj.nig.ac.jp/resource/bioproject/PRJDB15427.

## Ethics statement

The animal study was reviewed and approved by the Committee for Animal Experimentation at Waseda University.

## Author contributions

AH and SSh contributed to conception and design of the study. KI, AH, SSa, MS, and CR conducted experiments and analyzed the data. KI, HS and YL analyzed the gut microbiota composition. KI and AH performed the statistical analysis. AH wrote the first draft of the manuscript. KI, AH and SSh mainly contributed to manuscript revision. All authors contributed to manuscript revision, read, and approved the submitted version.
